# Quantitative Assessment of Abdominal Physical Features Associated with Cold Pattern

**DOI:** 10.3390/jcm15145485

**Published:** 2026-07-13

**Authors:** Keun Ho Kim, Jun-Su Jang, Seok-Jae Ko, Jae-Woo Park

**Affiliations:** 1 Korea Institute of Oriental Medicine, 1672 Yuseongdae-Ro, Yuseong-Gu, Daejeon 34054, Republic of Korea; junsu.jang@kiom.re.kr; 2 Kyung Hee University Hospital at Gangdong, 892 Dongnam-Ro, Gangdong-Gu, Seoul 05278, Republic of Korea; kokokoko119@hanmail.net (S.-J.K.); pjw2907@hanmail.net (J.-W.P.)

**Keywords:** cold pattern, abdominal examination, quantitative assessment, algometry, abdominal morphology, integrative medicine

## Abstract

**Background:** Abdominal examination (AE) is an important component of clinical assessment in Traditional East Asian Medicine (TEAM), providing information on abdominal shape, tenderness, stiffness, and skin color. However, conventional AE relies largely on subjective judgment and lacks standardized quantitative indicators. This study aimed to quantitatively characterize abdominal physical features associated with the cold pattern (CP) using objective abdominal examination devices and to explore their potential role in supporting standardized pattern-related assessment. **Methods:** A case–control study was conducted including 63 patients with functional dyspepsia and 60 healthy controls. Participants were classified according to CP status using a validated cold–heat pattern identification questionnaire. Abdominal features were quantified using a digital algometer and a depth-based geometric assessment system, measuring algometric (pressure tolerance, indentation depth, stiffness), geometric (abdominal depth and curvature), and chromatic (CIE L*a*b*) parameters. Group differences were analyzed using generalized linear models adjusted for confounders. A two-stage LASSO logistic regression with nested 10-fold cross-validation was applied. **Results:** Individuals with CP showed significantly lower pressure tolerance, indentation depth, stiffness, and CIE a* values, along with a flatter abdominal contour. The integrated model achieved a cross-validated ROC–AUC of 0.777 (95% CI, 0.674–0.872), indicating moderate discriminative performance. **Conclusions:** Quantitative algometric, geometric, and chromatic abdominal features were significantly associated with CP. Objective abdominal measurements may complement conventional AE by providing quantitative physical indicators that support more standardized and clinically relevant pattern-related assessment. These findings highlight the potential clinical utility of quantitative abdominal evaluation in improving diagnostic consistency. Trial registration: KCT0003369. Registered 23 November 2018.

## 1. Introduction

Pattern identification (PI) is a fundamental diagnostic framework in Traditional East Asian Medicine (TEAM), integrating clinical symptoms, physical signs, and individual characteristics to guide treatment decisions [1,2,3,4,5,6]. Within this framework, the cold–heat pattern (CHP) represents one of the most widely applied and clinically meaningful dimensions, reflecting differences in physiological status such as circulation, metabolic activity, and tissue characteristics. In clinical practice, the cold pattern (CP) is typically associated with features such as cold intolerance, reduced metabolic activity, and pale complexion, whereas the heat pattern (HP) is characterized by the opposite tendencies [7,8,9,10,11].

Despite its clinical importance, PI remains largely dependent on practitioners’ subjective interpretation, which limits its reproducibility and standardization. To address this limitation, previous studies have attempted to objectify CHP-related characteristics using various measurement approaches, including thermal imaging, pulse waveform analysis, and tongue image analysis [12,13,14,15]. Although these approaches have contributed to improving the objectivity of pattern-related assessment, many still rely on indirect or self-reported indicators, and discrepancies between subjective symptoms and objective physical findings have been reported. Therefore, there remains a need for additional objective and reproducible physical indicators that can complement conventional pattern identification. Such indicators may also facilitate more consistent patient stratification and improve the reproducibility of pattern-related clinical decision-making.

In this context, the identification of reliable physical indicators from specific body regions has attracted increasing attention. Previous studies have suggested that cold–heat sensations in different body regions may reflect distinct physiological mechanisms. In particular, abdominal cold–heat sensations have been reported to show weaker associations with conventional metabolic indicators compared to those in the extremities, suggesting that abdominal features may represent unique physiological characteristics [16].

Among physical examination methods in TEAM, abdominal examination (AE) is particularly well suited for assessing pattern-related physiological states. AE is a core component of physical assessment in TEAM and provides clinically relevant information regarding abdominal shape, tension, tenderness, resistance, and skin color, which are frequently used to inform pattern-based clinical decision-making [17,18,19]. In traditional practice, practitioners assess abdominal stiffness using their fingertips (typically the second, third, and fourth fingers) and evaluate abdominal shape and color through manual inspection. However, such approaches rely heavily on tactile perception and visual inspection, leading to substantial inter-examiner variability and limited reproducibility.

Recent technological developments, including abdominal examination devices (AEDs) and international standardization efforts such as ISO/TS 20758:2019, have enabled the objective quantification of abdominal physical features [20,21,22,23,24,25,26,27,28]. Digital algometers allow standardized evaluation of pressure-related sensitivity and tissue resistance, while depth-based imaging systems provide quantitative information on abdominal geometry and surface characteristics.

Previous studies have demonstrated that abdominal physical characteristics, including tenderness, stiffness, thermal properties, geometry, and color, can be quantitatively assessed using objective measurement techniques [20,21,22,23,24,25,26,27,28]. These abdominal features may provide clinically relevant information regarding physiological and pattern-related conditions. However, most previous investigations have focused on individual measurement modalities, and integrated analyses combining multiple domains of abdominal features remain limited. In particular, the potential of multimodal quantitative abdominal assessment to characterize CP-related physical features within a unified analytical framework has not been sufficiently explored. The clinical significance of the abdominal region is well recognized in TEAM. In traditional theory, the upper abdominal region is considered to reflect the functional state of the Middle Burner (Zhong Jiao), which is associated with digestion, absorption, and the regulation of qi and blood [29]. Accordingly, the upper abdomen has long been regarded as an important region for pattern-related assessment and clinical decision-making.

From an integrative clinical perspective, objective and reproducible physical indicators may contribute to more standardized assessment and improved consistency in pattern-related diagnosis. In addition, such quantitative approaches may support the development of complementary diagnostic tools that bridge traditional clinical insights with modern measurement techniques. Therefore, the present study aimed to quantitatively characterize abdominal physical features associated with CP using objective AEDs. By integrating algometric, geometric, and chromatic parameters, we sought to identify reproducible abdominal characteristics related to CP and to evaluate the feasibility of a multivariate analytical framework that may support more standardized and objective pattern-related assessment in clinical practice and research settings, with a focus on improving diagnostic consistency. We hypothesized that individuals with the cold pattern exhibit distinct quantitative abdominal characteristics and that the integration of algometric, geometric, and chromatic features would enable the development of a multivariable model capable of distinguishing CP from non-CP individuals. To address these objectives, an observational study was conducted in which participants were classified into CP and non-CP groups based on a validated questionnaire.

## 2. Materials and Methods

### 2.1. Participants

Participants were recruited between April 2018 and July 2020 at Kyung Hee University Hospital at Gangdong, Republic of Korea. The study population consisted of 63 adults diagnosed with functional dyspepsia who reported epigastric pain or discomfort. A comparison group of 60 healthy volunteers without gastrointestinal complaints was also included.

Prior to enrollment, all candidates underwent clinical screening to exclude significant systemic disorders involving major organs, including the cardiovascular, respiratory, hepatic, renal, and gastrointestinal systems. Individuals were not eligible for participation if they met any of the following criteria: previous gastrointestinal surgery; a history of psychiatric disorders; pregnancy or lactation; the presence of alarm symptoms such as unexplained weight loss, melena, or dysphagia; participation in another interventional study within 30 days before enrollment; confirmed human immunodeficiency virus infection; or any physical, cognitive, or social condition considered likely to interfere with study procedures or compliance, including severe sensory impairment, substance abuse, paralysis, or inability to complete study-related assessments.

**Figure 1:**
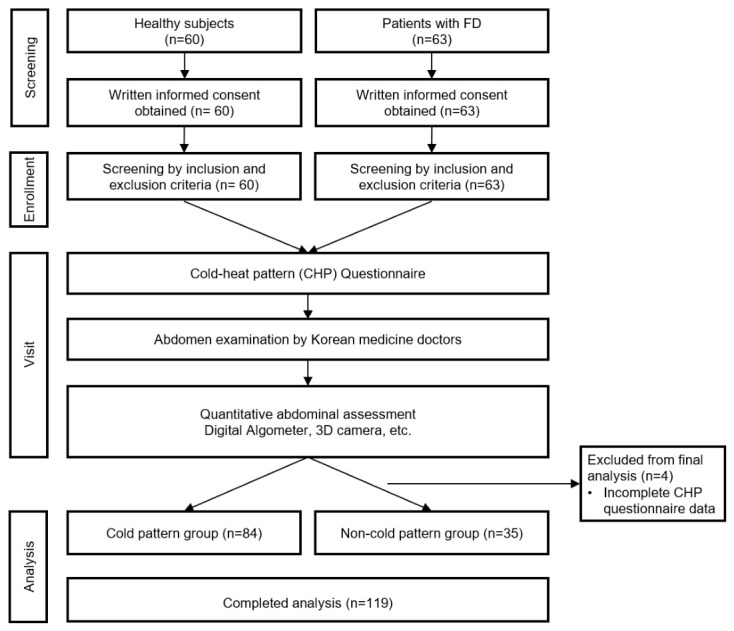
Study flow diagram of participant recruitment, exclusion, and classification.

The study protocol was approved by the Institutional Review Board of Kyung Hee University Hospital at Gangdong (KHNMCOH 2018-03-002-001) on 11 April 2018 and was conducted in accordance with Good Clinical Practice principles and applicable ethical guidelines. Written informed consent was obtained from all participants before any study-related procedures were performed. Eligibility to provide informed consent was verified prior to enrollment, and participation was entirely voluntary. Participant safety was monitored throughout the study in accordance with the approved protocol, and any reported adverse events were documented. All enrolled participants completed the study procedures. The participant recruitment, exclusion, and classification procedures are summarized in Figure 1. During data processing, four participants were excluded from the final analysis because of incomplete CHP questionnaire data.

### 2.2. Cold Pattern and Heat Pattern

In this study, multiple standardized questionnaires [22,30,31,32,33,34,35] were administered to assess gastrointestinal disorders and related symptoms. Special emphasis was given to CHP questionnaires, since differentiation between cold and heat manifestations constitutes a core diagnostic principle in TEAM [6,9].

CP and HP groups were classified using a standardized questionnaire for CHP identification, based on common symptoms in the general population [9,10]. The reliability and agreement of this questionnaire have been previously assessed [10,11]. The questionnaire comprises 15 items in total, with 8 items designated for CP classification and 7 for HP classification. Each item is rated on a 5-point Likert scale, ranging from 1 (strongly disagree) to 5 (strongly agree). The survey was completed by self-report. Participants were classified into CP and non-CP groups based on a predefined cutoff value of 21.5 for the CP score [10]. The grouping was determined according to the validated CHP questionnaire rather than disease status. Accordingly, participants from both the functional dyspepsia and healthy control cohorts were classified into the CP or non-CP group using the same predefined criteria. Similarly, participants were classified into the HP group if their HP item scores exceeded 17.5 for men and 16.5 for women; otherwise, they were assigned to the non-HP group [10]. Because cold and heat manifestations are not mutually exclusive in TEAM, individuals may exhibit characteristics of both HP and CP simultaneously. The questionnaire-based classifications were reviewed by experienced TEAM practitioners to ensure consistency with the study protocol and established diagnostic principles.

### 2.3. Abdominal Examination Devices and Measurement Protocol

**Figure 2:**
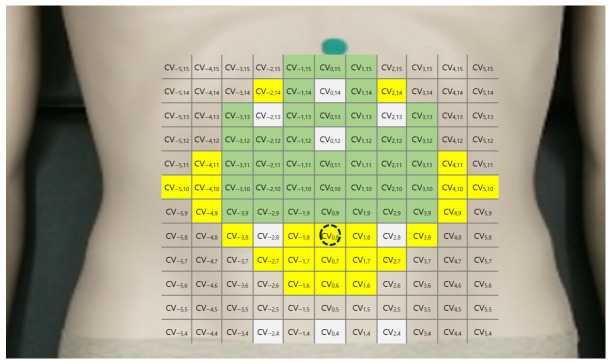
Anatomical locations of the measurable abdominal points used for quantitative abdominal examination. The dotted circle indicates the location of the umbilicus (CV_0,8_). The points were used for algometric, chromatic, and geometric assessments.

To obtain more objective indicators for abdominal diagnosis, advanced measurement techniques were incorporated. Abdominal color images were assessed using CIE L*a*b* values corrected with a color chart (ColorChecker, X-Rite, Grand Rapids, MI, USA) [36,37]. Abdominal geometry was captured with a three-dimensional (3D) camera (Kinect v2 (Microsoft, Redmond, WA, USA)) [38,39], while painful areas and resistance were quantified using a digital algometer [20]. These devices provided quantitative data, reducing reliance on visual inspection and palpation. The validity, repeatability, and measurement reliability of these devices have been previously evaluated. Previous studies reported high inter-trial reliability of the modified algometer (ICC = 0.849, 95% CI: 0.703–0.923) and demonstrated its diagnostic validity for assessing abdominal tenderness and stiffness [40]. In addition, repeated measurements of 3D geometric features yielded standard deviations ranging from 0.3 to 0.8 mm, while repeated measurements of color-corrected CIE L*a*b* values yielded standard deviations ranging from 0.5 to 1.5, indicating acceptable measurement reproducibility [23]. All measurements were conducted with participants in a supine position under standardized conditions. The upper abdomen was exposed, and ambient light was minimized to ensure consistent image acquisition. Abdominal measurement points were defined based on standardized acupuncture locations and proportional anatomical distances. The region between the xiphisternum and the umbilicus was divided into eight equal segments, each defined as 1 chon, and a grid of measurement points was established along the midline and bilateral regions. The spatial arrangement of measurement points is illustrated in Figure 2.

Depth images were acquired using a 3D camera and processed to obtain spatial information on abdominal surface characteristics, which were analyzed using a lattice-based grid. Color values were extracted from predefined abdominal regions (yellow, green, and white areas) for quantitative chromatic assessment. According to the Nan Jing [29], the upper abdominal region is considered important for evaluating cold–heat and deficiency–excess patterns. Accordingly, geometric depth measurements were obtained from selected upper abdominal regions (green and white areas).

Algometric measurements were performed at predefined abdominal sites using a digital algometer. The examiner applied pressure perpendicularly to the abdominal surface at a constant rate of 1 kg/cm^2^/s, guided by real-time visual feedback on a monitor to ensure consistent force application. Participants were instructed to press a buzzer when the applied pressure reached their subjective pain threshold, at which point pressure and indentation depth were automatically recorded and the operator ceased applying pressure. Measurements were repeated at nine designated abdominal sites. These sites were selected by synthesizing abdominal palpation regions described in Japanese and Korean traditional medicine literature [20,41,42], including representative acupoints such as CV14, CV12, ST21 (bilateral), ST25 (bilateral), KI16, and CV4. The final measurement regions were defined based on overlapping areas identified across these sources and correspond to the white regions shown in Figure 2. All measurements were performed by trained practitioners according to standardized procedures.

### 2.4. Calculation of Quantitative Physical Factors

Quantitative physical variables were derived from the abdominal color, geometric, and algometric measurements as described below. Abdominal morphology encompassed both color and geometric features. Color features were calculated as the mean CIE L*a*b* values within a 1 cm × 1 cm region centered at each predefined measurement point. The measurable color information corresponded to the yellow, green, and white regions shown in Figure 2, with components belonging to the CIE L*a*b* coordinate system. Geometric features included abdominal depth and depth differences between predefined measurement points, expressed in millimeters (mm). For clarity and consistency, simplified notations were used to represent bilateral and vertical depth differences. The bilateral depth difference, $Dp{t\_Diff\_CV}_{\left(x,-x\right),y}$, was defined as the difference in depth between symmetric left and right points at the same horizontal level, while the vertical depth difference, $Dp{t\_Diff\_CV}_{x,(y,y+1)}$, was defined as the difference in depth between adjacent points along the vertical axis. The mathematical definitions of these differences are provided in Equations (1) and (2), respectively.

(1) $$Dp{t\_Diff\_CV}_{\left(x,-x\right),y}=d({CV}_{x,y})-d({CV}_{-x,y})$$

(2) $$Dp{t\_Diff\_CV}_{x,(y,y+1)}=d({CV}_{x,y})-d({CV}_{x,y+1})$$

The bilateral and vertical depth differences defined in Equations (1) and (2) were calculated using predefined abdominal measurement coordinates located within the green and white regions shown in Figure 2.

Algometric variables included applied pressure ($Pr({CV}_{x,y})$ in kilogram-force [kgf]), the indented depth ($d({CV}_{x,y})$ in mm), and pressure per unit depth (stiffness, $St\left({CV}_{x,y}\right)$, expressed in kgf/mm). These values were recorded at the moment when participants reported pain at each predefined measurement point. Algometric measurements were obtained from the white regions shown in Figure 2.

(3) $$St\left({CV}_{x,y}\right)=\frac{Pr({CV}_{x,y})}{d({CV}_{x,y})}$$

### 2.5. Statistical Analysis for Identification of Significant Variables for CP

All statistical analyses were conducted using R software (version 4.0.0; R Foundation for Statistical Computing, Vienna, Austria). Statistical significance was defined as a two-sided p-value below 0.05. Baseline demographic and clinical characteristics were compared between the CP and non-CP groups. Continuous variables were evaluated using independent-sample *t*-tests, whereas categorical variables were examined using Fisher’s exact tests. To investigate associations between CP status and quantitative abdominal measurements, multivariable generalized linear models were fitted for each abdominal feature. Group membership (CP versus non-CP) was treated as the primary explanatory variable. Age, sex, body mass index (BMI), alcohol consumption, and caffeine intake were included as adjustment variables because of their potential influence on abdominal morphology and physiological characteristics. For each model, covariate-adjusted group estimates together with corresponding 95% confidence intervals were calculated. Differences between groups were interpreted on the basis of the adjusted model results.

### 2.6. Lasso Logistic Regression Analysis

A multivariable modeling approach was used to identify quantitative abdominal features associated with the cold pattern (CP). Candidate predictors included CIE L*a*b* color parameters, abdominal depth measurements, bilateral and vertical depth-difference variables, and algometric features such as pressure tolerance, indentation depth, and stiffness. CP status was defined as the binary outcome variable. Missing data were evaluated prior to analysis. No missing values were identified in the final analytical dataset; therefore, no imputation procedure was required. To reduce redundancy among predictors, pairwise correlations were examined, and when highly correlated variables were identified (|r| ≥ 0.90), only one representative variable was retained. All predictors were standardized to have a mean of zero and a standard deviation of one before model fitting.

Variable selection was performed using least absolute shrinkage and selection operator (LASSO) logistic regression. In the first stage, a LASSO model with 10-fold cross-validation was fitted to estimate regression coefficients (β). Predictors were ranked according to the absolute magnitude of their coefficients (|β|), and the ten variables with the largest |β| values were retained to improve model interpretability and reduce model complexity. In the second stage, a final LASSO logistic regression model was constructed using the selected predictors. The regularization parameter (λ) was optimized through cross-validation, and the value yielding the highest ROC–AUC was chosen.

To obtain an unbiased estimate of predictive performance, nested 10-fold cross-validation was employed, with the inner loop used for λ optimization and the outer loop used for model evaluation. Model performance was assessed using the area under the receiver operating characteristic curve (ROC–AUC). The 95% confidence interval for the ROC–AUC was calculated using the percentile bootstrap method based on 2000 resampling iterations. Variables with nonzero coefficients in the final model were considered important contributors to the multivariate characterization of CP-related abdominal features. The sign and magnitude of β were used to interpret the direction and relative strength of the association between each predictor and CP status.

## 3. Results

### 3.1. Comparison of General Characteristics

As shown in Figure 1, following exclusion of four participants with incomplete CHP questionnaire data, 119 individuals were included in the analysis, comprising 84 participants classified as the CP group and 35 as the non-CP group (Table 1). The study population consisted of 20 men and 99 women. Compared with the non-CP group, the CP group showed significantly higher age and significantly lower height, body weight, body mass index (BMI), and systolic blood pressure. Significant differences were also observed in sex distribution and alcohol consumption. In contrast, no significant between-group differences were found for diastolic blood pressure, pulse rate, body temperature, or caffeine intake.

### 3.2. Comparison Between Groups According to Abdominal Examination

Geometric, chromatic, and algometric abdominal features exhibited distinct patterns according to CP status. Algometric features, including pressure, indentation depth, and stiffness, tended to be lower in the CP group than in the non-CP group (Table 2), with mean differences ranging from −1.04 to −0.83 for pressure, −5.49 to −4.48 for depth, and −0.024 to −0.017 for stiffness. Geometric analyses identified several significant differences in abdominal depth-related variables (Table 3 and Table 4). In particular, multiple vertical depth-difference variables differed between groups, whereas no significant bilateral depth differences were observed. In addition, CIE a* values were lower in the CP group across several abdominal regions (Table 5), with mean differences ranging from −0.66 to −0.32. No other abdominal variables showed statistically significant between-group differences.

### 3.3. Regression Model

**Figure 3:**
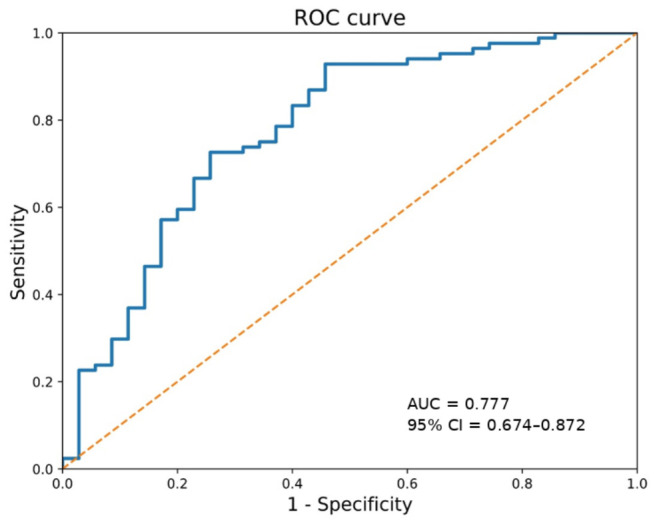
Receiver operating characteristic (ROC) curve of the final LASSO logistic regression model obtained using nested 10-fold cross-validation for discrimination between the CP and non-CP groups. The blue solid line represents the ROC curve of the final model, and the orange dashed line represents the reference line corresponding to random classification (AUC = 0.5). The model achieved a cross-validated ROC–AUC of 0.777 (95% CI, 0.674–0.872).

After multicollinearity reduction and LASSO-based variable selection, 10 predictors with the highest absolute regression coefficients were selected to construct the final multivariable model (Table 6). These predictors included color, geometric, and algometric features. The final LASSO logistic regression model achieved a cross-validated ROC–AUC of 0.777 (95% CI, 0.674–0.872), indicating moderate discriminative performance (Figure 3). Using a threshold selected to balance sensitivity and specificity, the model yielded a sensitivity of 0.726 and a specificity of 0.743. The nested cross-validation procedure was used to provide an internally validated estimate of model performance. The selected predictors included color, geometric, and algometric variables, indicating that multiple domains of abdominal features contributed to the final model.

## 4. Discussion

This study quantitatively characterized abdominal physical features associated with CP using objective AEDs. By integrating algometric, geometric, and chromatic parameters, we identified quantitative abdominal characteristics consistent with traditional descriptions of CP. Specifically, CP was associated with reduced pressure tolerance, decreased abdominal depth, and lower chromatic intensity, providing quantitative support for traditionally described abdominal findings.

**Figure 4:**
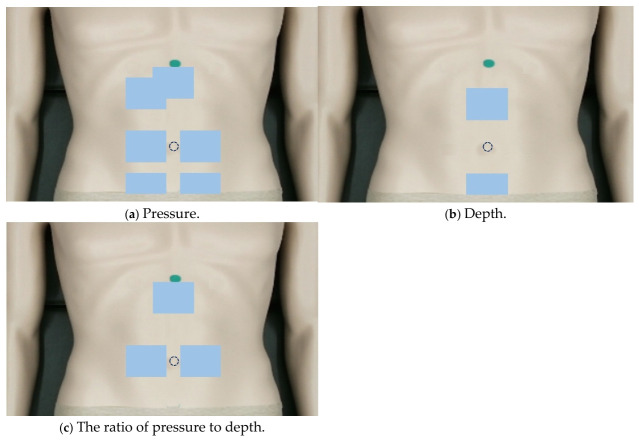
Abdominal regions showing significant differences in algometric features between the CP and non-CP groups. Colored regions indicate locations with statistically significant differences identified using generalized linear models adjusted for age, sex, BMI, alcohol consumption, and caffeine intake. Blue regions indicate lower values in the CP group than in the non-CP group. (**a**) Pressure, (**b**) indentation depth, and (**c**) pressure-to-depth ratio.

Algometric analysis showed that individuals with CP exhibited significantly lower pressure tolerance, indentation depth, and stiffness-related indices across multiple abdominal locations, as illustrated in Figure 4a, b, and c, respectively. These findings suggest reduced tissue stiffness and increased sensitivity to external pressure in individuals with CP. Digital algometers have been shown to reliably assess abdominal stiffness and tenderness. From a TEAM perspective, decreased abdominal resistance and increased tenderness are characteristic features of CP, and the present findings provide quantitative support for these traditional interpretations.

**Figure 5:**
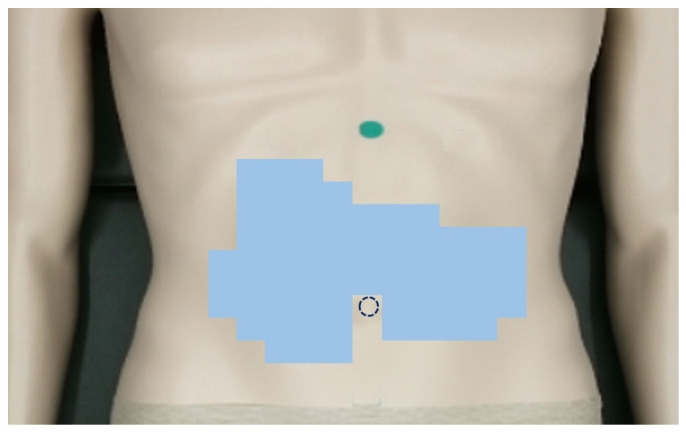
Abdominal regions showing significant differences in CIE a* color values between the CP and non-CP groups. Colored regions indicate locations with statistically significant differences identified using generalized linear models adjusted for covariates. Blue regions indicate lower CIE a* values in the CP group than in the non-CP group, corresponding to reduced abdominal skin redness.

Chromatic analysis demonstrated consistently lower CIE a* values across large abdominal regions in individuals with CP, indicating a paler abdominal skin appearance, as shown in Figure 5. As the a* component reflects the red–green axis and has been associated with skin redness, hemoglobin-related coloration, and superficial skin perfusion [43,44], the lower a* values observed in CP may indicate reduced abdominal blood perfusion. These findings support the potential physiological relevance of chromatic features as objective indicators of pattern-related characteristics.

**Figure 6:**
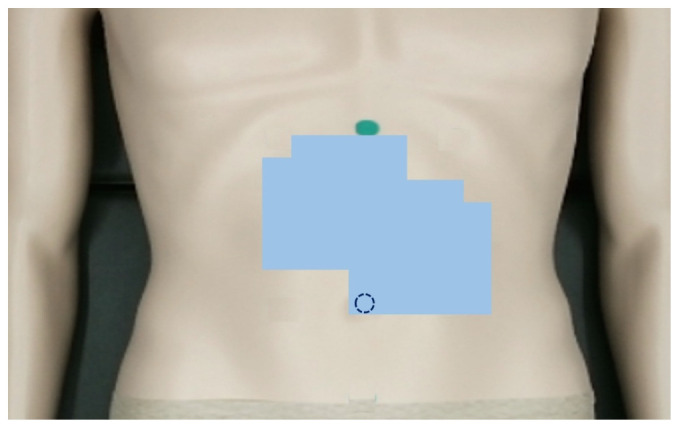
Abdominal regions showing significant differences in abdominal depth between the CP and non-CP groups. Colored regions indicate locations with statistically significant differences identified using generalized linear models adjusted for covariates. Blue regions indicate lower abdominal depth values in the CP group than in the non-CP group, corresponding to a flatter abdominal contour.

Geometric analysis using 3D imaging revealed reduced abdominal protrusion and vertical curvature in individuals with CP, particularly in the upper and central abdominal regions (Figure 6), suggesting a flatter abdominal contour that may reflect reduced muscle tone, decreased tissue elasticity, or diminished structural support.

Taken together, these findings may reflect physiological characteristics traditionally associated with CP. Reduced CIE a* values may be related to diminished peripheral circulation, whereas decreased abdominal protrusion may be associated with differences in abdominal morphology, tissue volume, or body composition. Similarly, lower pressure tolerance and stiffness-related indices may indicate changes in tissue elasticity and mechanical properties. Although these interpretations are biologically plausible and consistent with traditional descriptions of CP, the underlying physiological mechanisms were not directly assessed in the present study and therefore require further investigation using objective measures of circulation, metabolism, and body composition.

To integrate heterogeneous abdominal features while minimizing overfitting, a two-stage LASSO logistic regression model with nested 10-fold cross-validation was employed. The final model demonstrated moderate discriminative performance (ROC–AUC = 0.777; 95% CI, 0.674–0.872) (Figure 3). The selected predictors included algometric, geometric, and chromatic features, highlighting the complementary contributions of multiple abdominal domains. These results provide preliminary evidence that multivariate integration of quantitative abdominal features may be useful for characterizing CP-related physiological characteristics. However, additional validation in independent cohorts is required before clinical application can be considered.

The location-specific analyses presented in Table 2, Table 3, Table 4 and Table 5 enabled the characterization of spatial patterns of abdominal physical features associated with CP. By examining multiple abdominal locations, this approach identified regional variations in algometric, geometric, and chromatic characteristics that may reflect the physiological manifestations of CP. These location-specific findings provided candidate features for subsequent multivariable modeling, and selected features were further evaluated using LASSO regularization and nested cross-validation.

The present model was not intended to serve as a definitive diagnostic classifier but rather to evaluate the feasibility of an objective analytical framework for integrating multiple abdominal features associated with CP. The ability of the model to achieve moderate discriminative performance using a limited number of predictors supports the potential utility of quantitative abdominal assessment as an objective adjunct to traditional pattern identification in clinical practice.

From a clinical perspective, quantitative abdominal assessment may provide a practical framework for reducing examiner-dependent variability and improving the standardization of abdominal examination in TEAM. Because the proposed approach relies on objective measurements obtained using noninvasive assessment devices, it may be readily implemented in both clinical and research settings. Objective measurements of abdominal characteristics could facilitate more consistent clinical documentation, support practitioner training, and enhance the reproducibility of pattern-related assessments across institutions. In the longer term, such quantitative approaches may contribute to the integration of traditional diagnostic concepts with evidence-based clinical practice.

Several factors should be taken into account when interpreting the present findings. The study was conducted in a relatively small cohort, which may have reduced the representativeness of the observed associations. Furthermore, a formal a priori sample size calculation was not performed because the study was exploratory in nature. In addition, individuals with organic gastrointestinal disorders were not included, and therefore the identified abdominal characteristics may not fully reflect the broader spectrum of clinical conditions encountered in routine practice.

Another consideration relates to the classification of CP. Although CP status was determined using previously validated questionnaires and subsequently reviewed by experienced TEAM practitioners, pattern identification remains a complex construct that lacks a universally accepted objective reference standard. Consequently, some degree of classification uncertainty cannot be completely excluded. Furthermore, the unequal distribution of participants between the CP and non-CP groups, including a substantial difference in sex distribution, may have influenced model estimation. Although sex was included as an adjustment variable together with age, BMI, alcohol consumption, and caffeine intake, residual confounding cannot be completely excluded because abdominal morphology, skin color, pressure sensitivity, and body composition may differ according to sex. Future studies with more balanced sex distributions are warranted.

The predictive framework was developed and evaluated within a single dataset and was not tested using an independent external cohort. Although nested cross-validation was employed to reduce overfitting and provide a more robust estimate of predictive performance, the generalizability of the model across different populations remains to be established. Future investigations involving larger multicenter cohorts, independent validation datasets, and additional objective physiological measurements are warranted to validate the identified feature patterns and further clarify the clinical applicability, reproducibility, and generalizability of quantitative abdominal assessment for pattern-related evaluation.

Despite these limitations, this study contributes to the advancement of objective and standardized AE in TEAM. By translating qualitative palpation and visual observations into objective quantitative measures, this approach provides a foundation for standardized and evidence-based assessment of pattern-related abdominal characteristics. Integration of such quantitative methods with conventional diagnostic frameworks may facilitate improved clinical consistency, enhanced research reproducibility, and more precise pattern-oriented therapeutic strategies.

## 5. Conclusions

In summary, this study quantitatively characterized abdominal physical features associated with CP in TEAM using objective AE methods. Individuals with CP exhibited distinct algometric, geometric, and chromatic characteristics, including reduced pressure tolerance and stiffness, a flatter abdominal contour, and lower CIE a* values. The integration of these features demonstrated the feasibility of a multivariate framework for capturing CP-related abdominal characteristics, which may complement conventional AE by enabling more standardized and objective pattern-related assessments. These findings contribute to the modernization of AE in TEAM by translating qualitative observations into measurable indices. Further studies with larger and more diverse populations are needed to validate these findings and explore their broader clinical applicability.

## Figures and Tables

**Table 1:** General characteristics of subjects between CP and non-CP groups.

	CP Group	Non-CP Group	*p*-Value
Number of subjects	84	35	
Age (years)	41.26 ± 11.49	35.89 ± 9.58	**0.011**
Height (cm)	161.94 ± 6.77	164.95 ± 7.08	**0.037**
Weight (kg)	56.90 ± 8.88	64.24 ± 13.63	**0.005**
BMI (kg/m^2^)	21.65 ± 2.65	23.47 ± 3.94	**0.016**
SBP (mmHg)	114.26 ± 12.47	120.31 ± 15.54	**0.046**
DBP (mmHg)	70.17 ± 8.43	72.31 ± 9.29	0.243
Pulse rate per minute	77.17 ± 10.20	74.29 ± 10.21	0.166
Body temperature (℃)	36.34 ± 0.35	36.28 ± 0.39	0.411
Gender			**0.015**
Men	8 (9.5%)	12 (34.3%)	
Women	76 (90.5%)	23 (65.7%)	
Alcohol			**<0.001**
No	52 (61.9%)	9 (25.7%)	
Yes	32 (38.1%)	26 (74.3%)	
Caffeine			0.650
No	20 (23.8%)	7 (20.0%)	
Yes	64 (76.2%)	28 (80.0%)	

CP—cold pattern; BMI—body mass index; SBP—systolic blood pressure; DBP—diastolic blood pressure. The values represent mean ± standard deviation for continuous variables and the frequency (percentage) for categorical variables. The *p*-values for differences between CP and non-CP groups were from two sample *t*-test for continuous variables and Fisher’s exact test for categorical variables.

**Table 2:** Locations with statistically significant pressure, depth, and stiffness (defined as the pressure-to-depth ratio) at the onset of pain in patients with CP.

Variables	Location	CP Group	Non-CP Group	Mean Difference (95% CI)	*p*-Value
Pressure	CV_0,14_	2.98 (1.17, 6.45)	4.02 (2.05, 7.89)	−1.04 (−1.60, −0.49)	0.012
	CV_−2,13_	2.98 (0.96, 5.77)	3.90 (1.29, 7.16)	−0.92 (−1.45, −0.38)	0.030
	CV_−2,8_	2.70 (0.62, 5.14)	3.63 (1.43, 6.90)	−0.94 (−1.45, −0.43)	0.023
	CV_2,8_	2.77 (1.03, 5.91)	3.73 (1.37, 8.24)	−0.96 (−1.53, −0.39)	0.031
	CV_−2,4_	2.61 (0.87, 6.35)	3.44 (1.47, 6.93)	−0.83 (−1.36, −0.30)	0.050
	CV_2,4_	2.72 (0.68, 5.93)	3.69 (1.29, 7.85)	−0.97 (−1.57, −0.36)	0.038
Depth	CV_0,12_	31.95 (18.00, 46.00)	36.43 (20.00, 50.00)	−4.48 (−7.10, −1.85)	0.033
	CV_0,4_	35.71 (17.00, 50.00)	41.20 (21.00, 50.00)	−5.49 (−8.71, −2.26)	0.015
Stiffness	CV_0,14_	0.097 (0.043, 0.180)	0.114 (0.055, 0.213)	−0.017 (−0.030, −0.004)	0.046
	CV_−2,8_	0.071 (0.019, 0.153)	0.095 (0.050, 0.203)	−0.024 (−0.036, −0.011)	0.028
	CV_2,8_	0.070 (0.033, 0.146)	0.092 (0.046, 0.204)	−0.022 (−0.035, −0.009)	0.043

**Table 3:** Abdominal locations showing statistically significant differences in abdominal depth between CP and non-CP groups.

Location	CP Group	Non-CP Group	Mean Difference (95% CI)	*p*-Value
CV_0,15_	−10.26 (−14.74, −4.18)	−8.75 (−12.80, −2.59)	−1.51 (−2.40, −0.62)	0.021
CV_−1,14_	−8.54 (−16.09, 0.21)	−6.35 (−11.36, 0.90)	−2.18 (−3.42, −0.95)	0.008
CV_0,14_	−9.35 (−17.31, −3.82)	−7.25 (−12.74, −0.05)	−2.10 (−3.17, −1.04)	0.008
CV_−2,13_	−9.29 (−16.49, −0.93)	−7.12 (−16.17, −0.68)	−2.17 (−3.54, −0.80)	0.040
CV_−1,13_	−9.21 (−19.41, −3.09)	−6.96 (−16.02, 1.61)	−2.26 (−3.57, −0.94)	0.028
CV_0,13_	−8.72 (−21.47, −1.68)	−6.18 (−13.58, 2.40)	−2.54 (−3.89, −1.20)	0.011
CV_−2,12_	−11.19 (−20.92, −3.92)	−9.12 (−16.92, −2.10)	−2.07 (−3.37, −0.76)	0.031
CV_−1,12_	−9.68 (−23.37, −2.71)	−7.05 (−17.09, 1.17)	−2.63 (−4.09, −1.17)	0.017
CV_0,12_	−8.41 (−23.63, −0.12)	−5.75 (−16.84, 3.81)	−2.66 (−4.27, −1.04)	0.031
CV_1,12_	−8.97 (−21.59, −0.99)	−6.54 (−16.89, 4.82)	−2.43 (−4.13, −0.73)	0.045
CV_2,12_	−10.46 (−21.13, −0.57)	−7.98 (−17.96, 3.58)	−2.48 (−4.21, −0.75)	0.036
CV_−2,11_	−13.28 (−23.56, −7.08)	−11.02 (−19.21, −5.26)	−2.27 (−3.52, −1.01)	0.010
CV_−1,11_	−10.35 (−24.17, −3.05)	−8.06 (−19.42, −2.14)	−2.29 (−3.73, −0.84)	0.026
CV_1,11_	−9.89 (−21.66, −1.46)	−7.49 (−20.11, 2.75)	−2.41 (−3.99, −0.83)	0.034
CV_2,11_	−12.53 (−22.94, −5.01)	−10.03 (−19.81, −0.20)	−2.50 (−3.99, −1.01)	0.011
CV_3,11_	−17.18 (−27.37, −7.21)	−14.63 (−23.91, −5.05)	−2.55 (−4.33, −0.77)	0.019
CV_2,10_	−13.94 (−23.87, −7.37)	−11.32 (−19.27, −3.50)	−2.62 (−3.85, −1.38)	0.002
CV_3,10_	−18.89 (−29.69, −8.85)	−16.40 (−23.91, −8.02)	−2.49 (−3.98, −0.99)	0.012
CV_1,9_	−11.66 (−20.94, −5.52)	−9.45 (−18.72, −4.74)	−2.21 (−3.44, −0.99)	0.025
CV_2,9_	−13.90 (−25.08, −6.69)	−11.40 (−17.80, −5.39)	−2.51 (−3.62, −1.39)	0.003
CV_3,9_	−18.97 (−31.23, −10.63)	−16.60 (−23.14, −12.31)	−2.36 (−3.72, −1.00)	0.016

**Table 4:** Difference in abdominal depth between two locations.

Location	CP Group	Non-CP Group	Mean Difference (95% CI)	*p*-Value
Dpt_Diff_CV_−1,(14,15)_	−1.00 (−9.12, 8.39)	1.04 (−3.62, 9.00)	−2.04 (−3.28, −0.80)	0.017
Dpt_Diff_CV_2,(12,13)_	−1.98 (−8.15, 1.82)	−1.23 (−3.40, 1.83)	−0.76 (−1.35, −0.16)	0.009
Dpt_Diff_CV_−2,(10,11)_	−0.85 (−3.97, 2.77)	−1.42 (−3.73, 1.14)	0.57 (0.07, 1.07)	0.030
Dpt_Diff_CV_−1,(10,11)_	−0.89 (−4.30, 3.04)	−1.63 (−4.39, 1.22)	0.74 (0.21, 1.27)	0.022

$Dp{t\_Diff\_CV}_{x,(y,y+1)}$: depth difference between CV_x,y_ and CV_x,y+1_ along the vertical axis.

**Table 5:** Locations with statistically significant CIE a* values in the CP.

Location	CP Group	Non-CP Group	Mean Difference (95% CI)	*p*-Value
CV_−3,13_	−1.02 (−3.45, 1.06)	−0.46 (−2.07, 1.51)	−0.56 (−0.88, −0.24)	0.044
CV_−3,12_	−1.26 (−3.88, 1.47)	−0.76 (−2.31, 0.12)	−0.49 (−0.79, −0.20)	0.033
CV_−2,12_	−1.29 (−4.00, 2.85)	−0.85 (−2.34, 0.40)	−0.45 (−0.76, −0.13)	0.050
CV_−3,11_	−1.31 (−3.59, 1.93)	−0.84 (−2.42, 0.43)	−0.47 (−0.76, −0.17)	0.043
CV_−2,11_	−1.35 (−3.80, 0.78)	−0.84 (−2.42, 0.88)	−0.51 (−0.83, −0.19)	0.022
CV_1,11_	−1.55 (−4.05, 1.16)	−1.13 (−2.33, 0.05)	−0.42 (−0.74, −0.10)	0.036
CV_−3,10_	−1.26 (−3.57, 1.33)	−0.84 (−2.37, 0.93)	−0.42 (−0.72, −0.11)	0.026
CV_−2,10_	−1.27 (−3.63, 1.29)	−0.75 (−2.60, 1.01)	−0.52 (−0.86, −0.17)	0.010
CV_1,10_	−1.49 (−3.69, 1.39)	−1.07 (−2.84, 0.47)	−0.43 (−0.76, −0.09)	0.029
CV_3,10_	−0.96 (−3.14, 1.69)	−0.55 (−1.82, 1.18)	−0.41 (−0.72, −0.10)	0.020
CV_4,10_	−1.06 (−3.25, 1.89)	−0.73 (−1.94, 1.66)	−0.32 (−0.64, −0.01)	0.047
CV_−4,9_	−1.46 (−3.35, 1.98)	−1.07 (−2.08, 0.22)	−0.39 (−0.68, −0.10)	0.033
CV_−3,9_	−1.35 (−3.54, 1.56)	−0.72 (−2.24, 1.07)	−0.63 (−0.96, −0.30)	0.002
CV_−2,9_	−1.44 (−3.53, 1.39)	−0.92 (−2.69, 1.24)	−0.52 (−0.89, −0.16)	0.015
CV_2,9_	−1.32 (−3.32, 1.50)	−0.71 (−2.23, 1.07)	−0.61 (−0.97, −0.25)	0.004
CV_3,9_	−1.04 (−3.01, 1.99)	−0.46 (−1.83, 1.20)	−0.58 (−0.92, −0.24)	0.003
CV_4,9_	−1.08 (−3.00, 2.02)	−0.69 (−1.79, 1.51)	−0.39 (−0.72, −0.05)	0.035
CV_−3,8_	−1.40 (−3.69, 1.16)	−0.85 (−2.36, 1.43)	−0.56 (−0.94, −0.17)	0.016
CV_−2,8_	−1.69 (−4.19, 0.59)	−1.04 (−2.88, 0.92)	−0.66 (−1.04, −0.27)	0.002
CV_2,8_	−1.39 (−3.43, 1.85)	−0.91 (−2.32, 1.09)	−0.48 (−0.84, −0.11)	0.014
CV_3,8_	−1.05 (−3.30, 1.49)	−0.55 (−1.95, 1.72)	−0.50 (−0.86, −0.14)	0.016
CV_−2,7_	−1.39 (−3.97, 0.95)	−0.85 (−2.63, 1.30)	−0.54 (−0.92, −0.16)	0.029

**Table 6:** LASSO logistic regression model with Top 10 predictors.

	Feature	Location	Coefficient	OR
Color and geometry	CIE a*	CV_3,9_	−2.93	0.053
		CV_−3,9_	−2.90	0.055
	Bilateral depth difference	Dpt_Diff_CV_(1,−1),15_	−3.15	0.043
		Dpt_Diff_CV_(1,−1),9_	−2.79	0.061
	Vertical depth difference	Dpt_Diff_CV_0,(12,13)_	3.64	38.01
		Dpt_Diff_CV_−1,(10,11)_	2.90	18.26
		Dpt_Diff_CV_−1,(12,13)_	−2.87	0.056
		Dpt_Diff_CV_−2,(11,12_)	−2.63	0.072
Algometric	Depth	CV_0,14_	−3.33	0.036
		CV_0,12_	−2.66	0.070

## Data Availability

The datasets generated during the current study are available via the Zenodo repository (https://doi.org/10.5281/zenodo.18399274) with restricted access due to ethical and privacy constraints. Access to the data may be granted upon reasonable request to the corresponding author.
